# Pathophysiological Mechanisms Underlying Idiopathic Normal Pressure Hydrocephalus: A Review of Recent Insights

**DOI:** 10.3389/fnagi.2022.866313

**Published:** 2022-04-28

**Authors:** Phillip A. Bonney, Robert G. Briggs, Kevin Wu, Wooseong Choi, Anadjeet Khahera, Brandon Ojogho, Xingfeng Shao, Zhen Zhao, Matthew Borzage, Danny J. J. Wang, Charles Liu, Darrin J. Lee

**Affiliations:** ^1^Department of Neurological Surgery, Keck School of Medicine, University of Southern California, Los Angeles, CA, United States; ^2^Keck School of Medicine, University of Southern California, Los Angeles, CA, United States; ^3^Laboratory of Functional MRI Technology, Stevens Neuroimaging and Informatics Institute, Keck School of Medicine, University of Southern California, Los Angeles, CA, United States; ^4^USC Neurorestoration Center, Keck School of Medicine, University of Southern California, Los Angeles, CA, United States; ^5^Department of Physiology & Neuroscience and the Zilkha Neurogenetic Institute, Keck School of Medicine, University of Southern California, Los Angeles, CA, United States; ^6^Division of Neonatology, Department of Pediatrics, Fetal and Neonatal Institute, Children’s Hospital Los Angeles, Los Angeles, CA, United States

**Keywords:** idiopathic normal pressure hydrocephalus (iNPH), glymphatic circulation, ventriculoperitoneal (VP) shunt, cerebral blood flow, dementia, communicating hydrocephalus, blood brain barrier (BBB) breakdown

## Abstract

The pathophysiologic mechanisms underpinning idiopathic normal pressure hydrocephalus (iNPH), a clinically diagnosed dementia-causing disorder, continue to be explored. An increasing body of evidence implicates multiple systems in the pathogenesis of this condition, though a unifying causative etiology remains elusive. Increased knowledge of the aberrations involved has shed light on the iNPH phenotype and has helped to guide prognostication for treatment with cerebrospinal fluid diversion. In this review, we highlight the central role of the cerebrovasculature in pathogenesis, from hydrocephalus formation to cerebral blood flow derangements, blood-brain barrier breakdown, and glymphatic pathway dysfunction. We offer potential avenues for increasing our understanding of how this disease occurs.

## Introduction

Idiopathic normal pressure hydrocephalus (iNPH) is a common dementia-causing neurological disorder seen in the elderly, with 120 new cases per year per 100,000 population greater than 70 years (Iseki et al., 2014; Martín-Láez et al., 2015). iNPH classically presents with the clinical triad of gait disturbance, urinary incontinence, and dementia, with gait disturbance typically presenting first and cognitive manifestations arising later. The hallmark of the disease is an enlarged ventricular system without an increase in intracranial pressure (ICP). Despite progress in characterizing iNPH and its natural history, its pathophysiology has not been clearly defined.

Treatment for iNPH is centered around cerebrospinal fluid (CSF) shunting, which leads to improvement in symptoms, including dementia, in many patients (Toma et al., 2013). This distinguishes iNPH from other causes of dementia which are largely irreversible and hence represents an opportunity to characterize mechanisms contributing to cognitive impairment. Ultimately, clinical responses to shunting are varied, and long-term outcomes indicate that while shunting improves the natural history, the durability of treatment is less than with other etiologies of hydrocephalus (Junkkari et al., 2021). Taken together, these data suggest that ventriculomegaly alone does not account for the natural history of iNPH, challenging the traditional role of CSF dynamics and appealing for a better understanding of iNPH’s underlying pathogenesis.

iNPH has been examined from many view points, ranging from intracranial pressure dynamics, traditional radiographic parameters, advanced neuroimaging modalities, analysis of regional and global cerebral perfusion, and changes at the cellular and molecular levels, including the activity of the glymphatic pathways and the blood-brain barrier. Conclusions to be drawn from this disparate body of work are unsettled. In this manuscript, we review recent evidence related to the pathophysiology of iNPH in the context of current theories, noting areas of interest for future study. In particular, we focus on changes involving the cerebrovasculature, which may be central to pathogenesis.

## Clinical Features

The diagnosis of iNPH involves a combination of clinical symptoms, radiologic findings, and results of diagnostic evaluations (Table 1; Relkin et al., 2005; Nakajima et al., 2021). The criteria for probable iNPH differ somewhat between American/European and Japanese guidelines and are listed separately in Table 1. While both guidelines are accepted and used in practice, the broader radiographic criteria in the American/European guidelines may lead to a greater proportion of patients classified as probable iNPH (Andersson et al., 2017). Confounding the work-up of iNPH is the lack of specificity in diagnostic criteria and overlapping features shared with other neurodegenerative conditions including Alzheimer’s disease and other movement disorders including Parkinsonism. Gait disturbance is the most common feature and typically is the first symptom to arise (Hebb and Cusimano, 2001).

**Table 1:** American/European (Relkin et al., 2005) and Japanese (Nakajima et al., 2021) criteria for probable idiopathic normal pressure hydrocephalus.

	*American/European*	*Japanese*
Clinical	Gait/balance disturbance One impairment involving either cognition or urination	More than one symptom in the clinical triad: gait disturbance, cognitive impairment, and urinary incontinence
Historical	Insidious onset of symptoms with progression over time Age >40 years at symptom onset Symptom duration for at least 3–6 months No previous insult which could lead to secondary hydrocephalus No other neurologic, psychiatric, or medical cause for symptoms	Age ≥60 years No obvious preceding diseases causing ventricular dilation (e.g., subarachnoid hemorrhage, meningitis, head injury) Clinical symptoms not completely explained by other neurological or non-neurological disease.
Investigational	Ventricular enlargement without macroscopic obstruction with Evans Index >0.3 At least one of the following features: 1. Enlargement of temporal horns without hippocampal atrophy 2. Callosal angle of ~90 degrees or less 3. Evidence of altered brain water content including periventricular signal changes 4. Aqueductal or fourth ventricular flow void seen on MRI CSF opening pressure on lumbar puncture between 5–18 mmHg (70–245 mmH_2_O)	Ventricular enlargement with Evans Index >0.3 CSF opening pressure ≤200 mm H_2_O, normal CSF content One of the following two features: 1. Neuroimaging features of narrowing of the sulci and subarachnoid space over the high convexity/midline surface (DESH) with gait disturbance: small stride, shuffle, instability during walking, and increase in instability on turning 2. Improvement of symptoms after CSF tap test and/or drainage test

**Figure 1:**
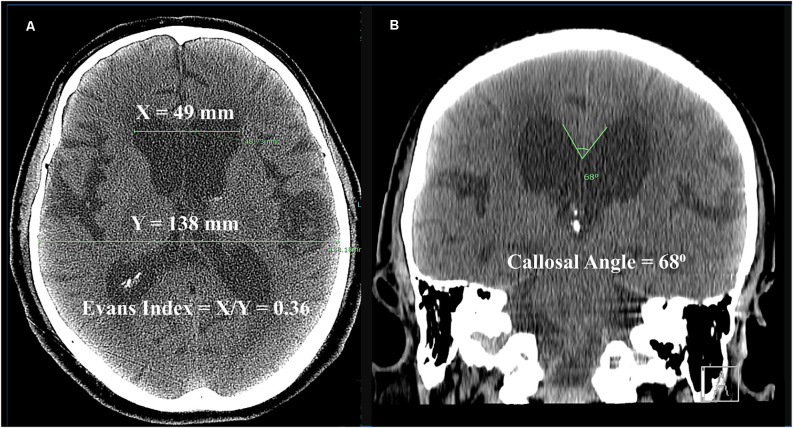
Computed tomography (CT) scan from a 70-year-old man with iNPH. **(A)** Axial image demonstrating Evans Index, which is the ratio of the maximum bifrontal horn width (X) to the maximum biparietal internal diameter of the skull (Y). An Evans Index >0.3 is present in iNPH. **(B)** Coronal image demonstrating callosal angle, here measured to 68 degrees. Normal callosal angles are greater than 90 degrees, while acute callosal angles occur in iNPH. Disproportionately enlarged subarachnoid space hydrocephalus (DESH) is apparent, as the Sylvian fissures are dilated out of proportion to sulci near the convexity.

Diagnostic radiographic features include ventricular enlargement with an Evans index of 0.3 or greater (Figure 1; Jacobs and Kinkel, 1976; George et al., 1995). Other common findings on brain imaging include a callosal angle of 90 degrees or less (Borzage et al., 2021), periventricular hyperintensities, and enlargement of the temporal horns (Relkin et al., 2005). A trial of CSF drainage is often undertaken to aid in the diagnosis, commonly *via* the lumbar subarachnoid space. Clinical benefit after temporary CSF drainage is strongly predictive of improvement in at least one symptom after shunting (Marmarou et al., 2005; Toma et al., 2013). In patients unable or unwilling to receive a shunt, serial lumbar punctures may be a treatment option (Isik et al., 2019).

Frustrating the iNPH clinical picture, there is not a clear neuroanatomic basis for the condition’s manifestations. It was originally held that stretching of white matter tracts from ventricular dilation led to iNPH’s clinical findings (Hakim and Adams, 1965), but there are several limitations with this hypothesis. First, it is not immediately obvious which white matter tracts would be causative. While the corticospinal tracts are clearly at risk for stretch, corticospinal tract-related gait disorders tend to differ from the hypokinesia and disequilibrium typical of iNPH (Rubino, 2002; Bugalho and Guimarães, 2007; Baker, 2018). Second, ventricular volume prior to CSF diversion-a surrogate for white matter tract stretching-does not correlate with the degree of symptomatology (Neikter et al., 2020). Third, other causes of ventriculomegaly both pathological (e.g., obstructive hydrocephalus) and physiological (e.g., ex vacuo hydrocephalus), do not typically cause gait disturbance as a primary manifestation. Fourth, the actual decrease in ventricular size after shunting is fairly modest in clinical responders, typically resulting in less than a 10% reduction in Evan’s index (Neikter et al., 2020). Thus, while changes in the corticospinal tracts and corpus callosum correlating with iNPH have been noted in some studies using tractography and transcranial magnetic stimulation (Röricht et al., 1998; Mataró et al., 2007; Siasios et al., 2016; Sarica et al., 2021), it is difficult to correlate symptomatic improvement following CSF drainage with decreased stretching of white matter tracts alone, and these changes may ultimately be epiphenomena.

Some authors have hypothesized that dysfunction of the cortico-basal ganglia-thalamo-cortical (CBGTC) is involved in iNPH (Curran and Lang, 1994; Lenfeldt et al., 2008), though the CBGTC loop may not explain dementia and urinary incontinence. Recent investigations have implicated striatal dopamine reuptake transporter density in iNPH gait impairment, offering a potential mechanism for basal ganglia dysfunction (Pozzi et al., 2021; Todisco et al., 2021). Other research has implicated frontal lobe dysfunction in not only the cognitive and urinary disturbances, but also gait disorder as well (Bugalho and Guimarães, 2007). As multiple lesional effects are suggested in iNPH, a fruitful approach to understanding the neuroanatomic basis may involve network-level investigations of functional connectivity (Griffa et al., 2020).

## Theories on Pathogenesis

Given shared features with other neurodegenerative conditions, iNPH is defined as a unique entity by ventricular enlargement. Our efforts to understand the etiology of the disease are thus inextricably tied to understanding how hydrocephalus develops in iNPH patients.

Generally speaking, communicating hydrocephalus in iNPH and other etiologies results from an imbalance between CSF formation and removal. This is thought to be due to impairment in the return of CSF to the circulation in most cases, through scarring or obstruction of the arachnoid granulations (Chen et al., 2017). A compelling anecdote to challenge this trad itional model is the observation that subarachnoid spaces are not universally dilated in communicating hydrocephalus (Egnor et al., 2002; Greitz, 2004). It warrants mention that in chronic states of hydrocephalus, CSF absorption is coupled to production; that is, while hydrocephalus may initially occur because of impaired absorption of CSF, the imbalance is a transient phenomenon, and the compensated state implies that a new equilibrium between absorption and production is reached. Theoretically, the new equilibrium may be reached through increased CSF absorption through other means, or by decreased CSF production.

Unlike other types of communicating hydrocephalus, such as subarachnoid hemorrhage, a lesion causing impaired CSF outflow is not apparent in iNPH. An alternative explanation of communicating hydrocephalus, however, invokes arterial pulsations. In this theory, homeostasis of the CSF spaces including the ventricles relies on the normal propagation through the cerebrovasculature of pulsations delivered through the cardiac cycle. In cases in which cerebral arteries lose compliance, the additional pulse pressure is delivered distally to the capillaries and veins, which may alter CSF dynamics in such a way to produce ventriculomegaly (Egnor et al., 2002; Greitz, 2004). Preliminary evidence suggests that CSF drainage may improve vascular compliance and, subsequently, CBF (Bateman, 2000). Hence, as there is not a lesion to block CSF egress at the level of the arachnoid granulations in the traditional model of communicating hydrocephalus, impaired vascular compliance may be sufficient to produce iNPH.

Pulsations transferred through the elastic arterial system cause movement of CSF back and forth through the aqueduct during the cardiac cycle (Marmarou et al., 1978; Linninger et al., 2005; Kahlon et al., 2007; Scollato et al., 2008; Ringstad et al., 2015; Yamada et al., 2020). In healthy adults the net movement of ventricular CSF is craniocaudal, however, in iNPH patients, the net movement is typically reversed, towards the third and lateral ventricles (Kim et al., 1999; Penn et al., 2011; Ringstad et al., 2016), which results in transependymal flow of ventricular CSF into the interstitial space (Ringstad et al., 2017). The flow pattern often reverts to anterograde flow after shunting (Ringstad et al., 2016). Clinical study of CSF dynamics in iNPH indicates elevated resistance to CSF outflow and increased CSF pulsatility. These features predict treatment response after CSF diversion, indicating normalization of CSF dynamics and a more physiologic state (Eide and Sorteberg, 2008, 2010; Malm et al., 2011; Qvarlander et al., 2013; Jacobsson et al., 2018).

**Figure 2:**
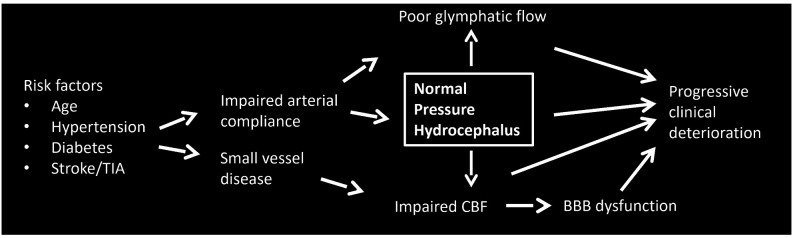
Flow diagram demonstrating possible pathogenic relationships in iNPH.

That iNPH may fundamentally represent a vascular disorder is intriguing, given the high incidence of vascular risk factors including hypertension and diabetes in iNPH patients (Eide and Pripp, 2014; Jaraj et al., 2016; Israelsson et al., 2017). Supporting this notion is the near-ubiquitous finding of deep white matter and periventricular lesions in iNPH (Krauss et al., 1997), hallmarks of small vessel disease. Variations in regional hypoperfusion and degree of hypoxic changes may help explain the clinical heterogeneity of iNPH and poor responses to shunting. Our view is that iNPH is fundamentally a cerebrovasculature disorder. Impaired compliance triggers a cascade of events culminating in the development of hydrocephalus, which subsequently begins a cycle that unchecked eventually progresses to irreversible dementia and neurologic injury. Shunting reverses some of the clinical manifestations, although even with treatment the disease is associated with progressive morbidity, which suggests a component of irreversible small vessel disease. Below we discuss three interrelated systems that may be central to the development and progression of iNPH: cerebral blood flow, the glymphatic system, and the blood-brain barrier. A flow diagram demonstrating possible pathogenic relationships is depicted in Figure 2.

## Deficient Cerebral Blood Flow (CBF)

Our understanding of the role of cerebral blood flow (CBF) in iNPH has evolved considerably in the last two decades. In early work, consistent patterns could not be drawn between the association between various CBF changes and: (1) the diagnosis of iNPH, (2) disease severity, or (3) improvement after shunting, which may have been due in part to disparate imaging protocols, inconsistent diagnostic criteria, and small sample sizes (Owler and Pickard, 2001). More recently, several studies reported regional hypoperfusion in critical areas, suggesting that vascular insufficiency is relevant to iNPH.

A number of studies have found regional CBF deficits in iNPH patients compared to age-matched healthy controls, including deficits in the periventricular white matter (Momjian et al., 2004; Ziegelitz et al., 2014, 2016; Virhammar et al., 2017), lentiform nucleus (Owler et al., 2004a; Ziegelitz et al., 2014, 2016; Virhammar et al., 2017), thalamus (Owler et al., 2004a; Virhammar et al., 2017), caudate (Owler et al., 2004a), and basal medial frontal cortex (Ziegelitz et al., 2014). Global reductions have been identified as well (Momjian et al., 2004; Owler et al., 2004a; Ziegelitz et al., 2014, 2016). One study noted inverse correlations between thalamic and putaminal CBF and severity of iNPH (Owler et al., 2004a), but most studies found no association between the magnitude of global or regional CBF values and severity of iNPH.

Clinical improvements after CSF drainage have been associated with improvements in CBF in the lateral and frontal white matter regions (Virhammar et al., 2014), periventricular white matter (Ziegelitz et al., 2015, 2016; Satow et al., 2017), periventricular thalamus (Ziegelitz et al., 2015), medial frontal region (Klinge et al., 2008), supplemental motor area (Lenfeldt et al., 2008), brainstem (Agerskov et al., 2020), and globally (Chang et al., 2009). Relating to prognostic variables prior to treatment, one study found decreased preoperative CBF in basal frontal lobes and anterior cingulate region in iNPH patients who responded to shunting compared to non-responders (Murakami et al., 2007). However, in most studies, no associations were found between preoperative regional CBF values and clinical response to shunting.

In light of this body of work, it is useful to consider the clinical findings in iNPH as the result of hypoperfusion. Depending on the extent of the CBF deficit, hypoperfusion may account fo rany or all findings of iNPH. Bladder dysfunction in iNPH is typically referable to detrusor overactivity, which may occur through effects on the frontal lobe or basal ganglia (Andersson, 2004; Sakakibara et al., 2008, 2012). Similarly, both the frontal lobe and basal ganglia have been implicated in gait disturbances that characterize iNPH (Bugalho and Guimarães, 2007). While cognitive impairment may be considered a diffuse lesion, some evidence suggests early frontal involvement in iNPH, consisting of psychomotor slowing and impaired attention rather than memory deficits, before progressing to more profound impairment (Iddon et al., 1999; Ogino et al., 2006; Picascia et al., 2015). With improved perfusion after shunting, symptoms may regress unless infarcts have already occurred.

The mechanism of impaired perfusion of deep gray matter and periventricular white matter is a subject of debate. Hypoperfusion may result from compression of the deep vascularity, compression of superficial venous outflow, impaired autoregulation, changes related to transependymal flow, or some combination of these factors (Momjian et al., 2004; Owler et al., 2004b; Bateman, 2008; Scollato et al., 2008; Chang et al., 2009). While a shunt would potentially improve any of these factors, the manner by which improvement in CBF occurs after shunting has not been well characterized. The effect of CSF drainage on cerebral perfusion pressure (CPP) through decreased ICP is limited as the ICP is not elevated, raising questions as to whether improved CPP alone may explain CBF changes (Eide and Sorteberg, 2010). One explanation may involve the CSF pulsatility curve, in which a relatively small change in ICP leads to decreased CSF pulsatility which may have downstream effects on CBF (Qvarlander et al., 2013).

## Impaired Glymphatic Circulation

**Figure 3:**
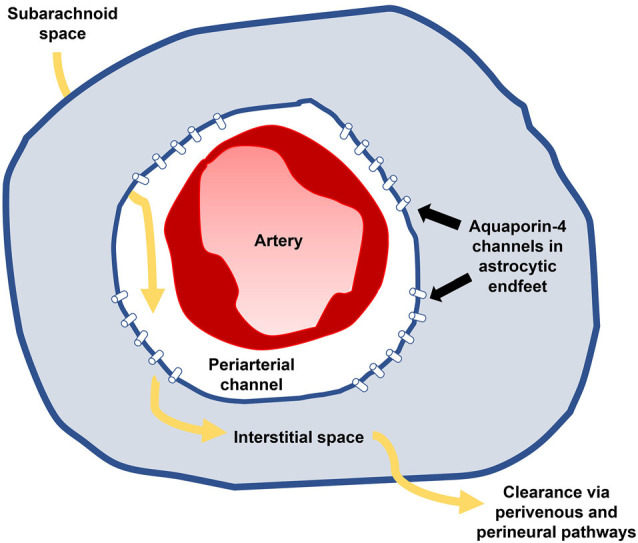
Glymphatic influx (yellow arrows) occurs along periarterial channels within subarachnoid spaces (white) and enters the parenchyma (blue) through aquaporin-4 transporters on astrocytic endfeet. Subarachnoid CSF joins interstitial fluid and passes through the brain, delivering substances to and from the parenchyma before being absorbed along perivenous and perineural channels. Impaired glymphatic circulation may result in part from impaired influx through poor arterial compliance and results in progressive neurotoxicity contributing to iNPH’s clinical manifestations.

The glymphatic (glial-lymphatic) system is a recently discovered homeostatic mechanism by which fluid moves through the brain parenchyma, acting as a waste disposal mechanism for brain tissue (Figure 3; Iliff et al., 2012; Nedergaard, 2013). In brief, subarachnoid CSF is pumped along periarterial channels by arterial pulsations (Iliff et al., 2013; Mestre et al., 2018) and enters the interstitial compartment through aquaporin-4 transporters within astrocyte endfeet (Plog and Nedergaard, 2018). CSF joins interstitial fluid and moves through the interstitial space towards perivenous and perineural channels through which it is removed from the brain. These efflux pathways include perisinusal lymphatic vessels that drain into extracranial lymphatics (Louveau et al., 2015; Ahn et al., 2019). Glymphatic circulation constitutes a primary role in the clearance of toxoids and waste products from the brain parenchyma, akin to the lymphatic function of other organs (Jessen et al., 2015), and is most active during sleep (Kang et al., 2009; Shokri-Kojori et al., 2018).

Mounting evidence suggests impairment of the glymphatic system by multiple mechanisms contributes to neurodegenerative diseases (Rasmussen et al., 2018). Much of the advances in understanding these pathways hail from the recognition that dysfunction of the glymphatic system contributes to amyloid-beta buildup in Alzheimer’s disease (Rasmussen et al., 2018; Mestre et al., 2020). Other conditions associated with glymphatic impairment relevant to iNPH include aging (Zhou et al., 2020), diabetes (Jiang et al., 2017), and hypertension (Mestre et al., 2018).

Clinical evaluation of iNPH patients demonstrates sluggish glymphatic flow (Ringstad et al., 2017; Eide and Ringstad, 2019; Bae et al., 2021). Lumbar intrathecal gadobutrol injection in iNPH patients resulted in delayed enhancement of subarachnoid spaces and cortical surfaces, compared to younger patients receiving gadobutrol for workup of intracranial hypotension (Ringstad et al., 2017). The age difference between the two patient groups somewhat limits the study’s conclusions, as even healthy older people have impaired glymphatic function (Zhou et al., 2020). Given that glymphatic dysfunction also is involved in Alzheimer’s disease (Tarasoff-Conway et al., 2015), this may represent a common pathway for cognitive decline in the two conditions (Reeves et al., 2020), but may not necessarily be related to urinary incontinence and gait in iNPH.

One theory posited to explain glymphatic impairment in iNPH is loss of arterial compliance. As vessels become increasingly stiff, the pump driving glymphatic influx is weakened, resulting in the buildup of waste substances in the interstitial fluid. This may in part explain the retrograde movement of subarachnoid CSF into the ventricular system, as the outflow resistance increases along glymphatic pathways. It is possible that the improvement in CSF dynamics after shunting (Ringstad et al., 2016) improves the glymphatic flow and thus cognition. However, that this could represent a primary insult in iNPH has been called into question (Gallina et al., 2020).

Another potential mechanism for glymphatic impairment is through reduced expression of aquaporin-4 channels, which has been demonstrated in iNPH patients (Hasan-Olive et al., 2019). In Alzheimer’s disease, decreased expression of aquaporin-4 leads to impaired clearance of misfolded proteins, which results in neurotoxicity and cognitive decline (Xu et al., 2015; Zeppenfeld et al., 2017). Glymphatic impairment in iNPH may lead to a similar buildup of waste products with resultant neurotoxicity that leads to cognitive dysfunction. Improvement in the glymphatic flow after shunting iNPH and subsequent clearance of accumulated interstitial substances may improve neuronal function and hence cognition after shunting, though this is untested.

## Blood-Brain Barrier Breakdown

**Figure 4:**
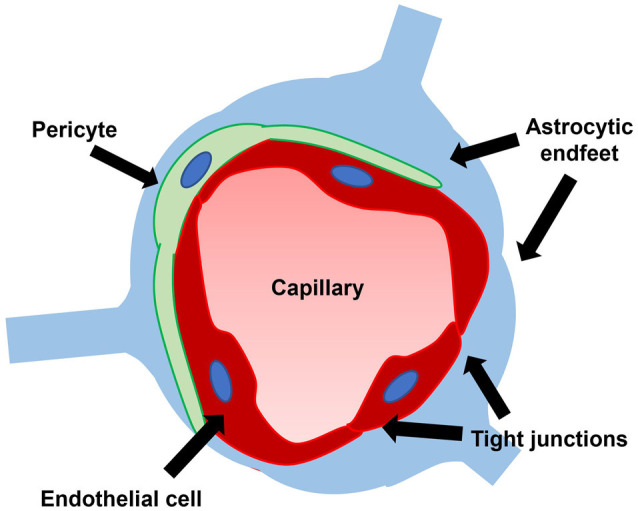
The blood-brain barrier consists of endothelial tight junctions, pericytes, and astrocytic endfeet. A leaky blood-brain barrier through dysfunction of one or multiple of these constituent parts has been demonstrated in iNPH.

The blood-brain barrier (BBB), the brain’s unique microvascular interface consisting of endothelial tight junctions, pericytes, and astrocytic endfeet, plays a critical role in maintaining the optimal conditions for proper neuronal functioning by acting as a selective barrier between the blood and brain (Figure 4; Bradbury et al., 1963; Bernacki et al., 2008; Abbott et al., 2010). The BBB prevents the entry of toxins while facilitating the transportation of metabolites and nutrients into the CNS. Given its role in homeostasis and neuroprotection, the BBB has been investigated in a host of neurological disorders (Sweeney et al., 2018; Profaci et al., 2020).

Several investigations have demonstrated pathology related to the BBB in iNPH patients. Distorted and thickened basement membranes and degenerated pericyte processes were demonstrated in biopsy specimens from iNPH patients (Eidsvaag et al., 2017; Eide and Hansson, 2020). Pericytes are essential components of the BBB and play important roles in induction, maintenance, and selective permeability (Abbott, 2002; Armulik et al., 2010). Pericyte degeneration has been shown to cause increased permeability to water and both low- and high-molecular-weight tracers, creating a leaky BBB (Armulik et al., 2010).

Additional evidence for compromise of the BBB was identified in iNPH patients through the extravasation of fibrin, a blood coagulation protein, in frontal biopsy specimens (Eide and Hansson, 2020). In this study, scattered fibrin staining around capillaries within cortical layers was seen in all iNPH patients compared to fewer than 30% of patients with other neurological diseases (Eide and Hansson, 2020). Increased fibrin deposition in the brain parenchyma in NPH biopsies correlated with increased levels of glial fibrillary acidic protein (GFAP), a marker of reactive astrogliosis (Eide and Hansson, 2020). Astrogliosis decreases compliance, which may in turn contribute to altered CSF dynamics in NPH (Lu et al., 2011; Fattahi et al., 2016). Both the degree of fibrin extravasation and astrogliosis correlated with reduced expression of aquaporin-4 transporters on perivascular astrocytic endfeet in biopsies of NPH patients, suggesting a link with glymphatic function (Eide and Hansson, 2018, 2020).

BBB dysfunction in iNPH may be related to deficient CBF. In a murine model of chronic cerebral hypoperfusion, hypoxia-induced injury to pericytes lead to BBB disruption (Liu et al., 2019). Though not tested in iNPH, a similar mechanism may explain pericyte injury and subsequent loss of BBB integrity. Pericyte dysfunction and other BBB insults have been demonstrated through means such as inflammation, hyperglycemia, and ischemia in Alzheimer’s disease, traumatic brain injury, and other disorders (Erickson and Banks, 2013).

## Conclusions

From the initial insult leading to hydrocephalus and onset of clinical manifestations to the irreversible changes occurring later in untreated cases, the cerebrovasculature is closely tied to the pathogenesis of iNPH. Relevant mechanisms include diminished CBF, glymphatic disruption, and changes to the BBB. Additional work is needed to further characterize how these pathophysiologic mechanisms inter-relate. Further, future studies should address how these pathologic features are reversed with shunting, which will provide insights into both iNPH and other neurodegenerative conditions. Answers to these questions will shed light on improving clinical responses and enhancing the durability of shunting.

## Author Contributions

PB, RB, KW, WC, and AK: investigation, manuscript writing, and editing. BO and XS: manuscript editing. ZZ, MB, and DW: conception and manuscript editing. CL and DL: conception, manuscript editing, and supervision. All authors contributed to the article and approved the submitted version.

## Conflict of Interest

The authors declare that the research was conducted in the absence of any commercial or financial relationships that could be construed as a potential conflict of interest.

## Publisher’s Note

All claims expressed in this article are solely those of the authors and do not necessarily represent those of their affiliated organizations, or those of the publisher, the editors and the reviewers. Any product that may be evaluated in this article, or claim that may be made by its manufacturer, is not guaranteed or endorsed by the publisher.
